# Special Issue “Chikungunya Virus and Emerging Alphaviruses”

**DOI:** 10.3390/v15081768

**Published:** 2023-08-19

**Authors:** Mylena R. Pereira, Rafael F. O. Franca

**Affiliations:** Fundação Oswaldo Cruz—Fiocruz/SP, Ribeirão Preto 14049-900, Brazil; myribeirop@gmail.com

Chikungunya virus (CHIKV), an alphavirus transmitted by mosquitoes, has experienced a recent re-emergence in various regions of the world, leading to large-scale outbreaks. In humans, CHIKV infection leads to a febrile illness known as chikungunya fever (CHIKF). Although most cases are self-limited, individuals may present prolonged joint pain and myalgia that can last for weeks or even months. In the most recent outbreaks, atypical manifestations of CHIKF have been documented, including severe arthralgia and neurological complications such as encephalitis, meningitis, and Guillain–Barré Syndrome. Tragically, fatalities have also been reported, with CHIKV now recognized as a significant public health concern. Several factors, such as virus evolution, globalization, and climate change, have likely contributed to the spread of CHIKV. One of the major challenges in combating CHIKV is the lack of effective preventive vaccines and approved antiviral treatments. This situation has escalated CHIKV to the status of a major global health threat. Addressing these challenges and investing in research and public health initiatives to control mosquito populations and develop effective interventions are crucial steps in mitigating the impact of CHIKV outbreaks on vulnerable populations worldwide. In this Special Issue, a total of 16 articles were published, addressing many aspects of CHIKV and other public health-relevant alphaviruses. This collection of articles contributes valuable insights into understanding the epidemiology, pathogenesis, clinical manifestations, and potential control strategies for CHIKV and related alphaviruses.

Out of the 16 published articles in this Special Issue, three of them are reviews that provide a comprehensive understanding of CHIKV and its different clinical manifestations. Interestingly, one of these articles describes the incidence of CHIKV cardiac manifestations. The authors conducted a systematic review and meta-analysis of data from the literature, revealing a global pooled incidence of cardiac involvement of 32.81% (95% CI 09.58–61.49, I2 = 96%) during CHIKV infections [1]. Two other reviews addressed the pathogenic mechanisms involved in CHIKV infection, providing updates on various aspects of CHIKV immunopathology as it presents in different organs [2], in addition to a highly cited review that covers numerous aspects of CHIKV pathology, epidemiology, and the current status of vaccine development [3]. The last published review provides an up-to-date perspective on antiviral development against Mayaro virus (MAYV), another alphavirus highly incident in the northern and central-western states of Brazil. This review not only addressed antiviral research but also provided a complete understanding of the MAYV virus structure, internalization, and replication. With respect to the antiviral aspects, the authors considered research employing synthetic molecules extracted from natural products as well as approved medicines already used to treat other diseases [4]. Thus, in this context, drug repurposing emerges as a promising strategy to combat MAYV and other viral infections.

Regarding epidemiological reports on CHIKV and other alphaviruses, three articles addressed the current aspects of CHIKV and other alphaviruses from different locations. Specifically, in Cameroon, between December 2019 and September 2021, there was a noted variation in the prevalence of chikungunya IgM. The prevalence of anti-CHIKV IgM ranged from 18.4% to 21.7% in Yaoundé and from 4.5% to 12.6% in Dizangué, as documented in the study [5]. Eastern equine encephalitis virus (EEEV) and Venezuelan equine encephalitis virus (VEEV) are zoonotic pathogens affecting humans, particularly equines. In Colombia, between 2008 and 2019, a total of 96 cases of EEE and 70 of VEE were reported, with 58% of EEE cases occurring in 2016 and 20% of EEV cases in 2013 [6]. In their 2022 study, Ribeiro et al. demonstrated that CHIKV outbreaks in the Amazon region were predominantly due to the circulation of the Asian Lineage. Furthermore, the authors identified a local transmission cluster linked to strains from the Caribbean and the Philippines [7]. Given the challenge of identifying co-circulating emerging arboviruses that cause infections with similar clinical symptoms in a population, the utilization of viral metagenomics for surveillance becomes vitally important. To address this challenge, the authors presented the findings from a metagenomic next-generation sequencing assay, tailored for use in samples where arboviral amplification is inconclusive [8].

In a cohort study conducted at the Bangkok Hospital for Tropical Diseases between 2019 and 2020, acute CHIKV-infected patients were evaluated to determine the relationship between viral load, clinical symptoms, and serological profiles. The findings revealed a significantly higher viral load in patients exhibiting symptoms of fever, headache, and arthritis. Phylogenetic analysis of the CHIKV strains under study identified them as belonging to the East, Central, and Southern African (ECSA) genotype, specifically of the Indian Ocean lineage (IOL) [9]. Severe neuropathies and neonatal infections can result from intrapartum vertical transmission. To investigate the correlation of intrahost genetic diversity and viral pathogenesis, the authors examined the intrahost genetic diversity of CHIKV from patients exhibiting neurological symptoms and in mothers infected during the intrapartum period, as well as in their neonates who acquired the infection through vertical transmission. No statistically supported differences were observed for the genetic variability (nucleotide substitutions/gene length) between the groups, suggesting that viral diversity is of limited consequence. This finding implies that other factors, potentially host-related or environmental, might play a more dominant role in determining the clinical outcomes of these specific CHIKV infections [10].

Significantly, the severity of CHIKV disease often correlates with viral persistence, potentially leading to chronic inflammation. In line with this, viral RNA was detected in semen specimens from six individuals with acute and post-acute CHIKV infections. The longest detection period extended to 56 days after the onset of disease symptoms [10,11]. Furthermore, the extended presence of viral RNA or antigens might intensify the production of pro-inflammatory mediators. A study providing an in-depth look into the inflammatory environment of infants exposed to maternal CHIKV discovered increased levels of the chemokines MIP-1α, MIP-1β, and CCL-2 (*p* < 0.05) as well as the cytokines TNFα, IL-6, and IL-7 (*p* < 0.0001) in CHIKV-exposed infants [12].

As of now, there are no licensed vaccines against CHIKV. Addressing this significant gap, researchers have constructed and preclinically characterized recombinant adenovirus vaccines expressing E2, E1, or E2-6K-E1 of CHIKV. These vaccine candidates demonstrated promising outcomes by inducing robust levels of CHIKV neutralizing antibodies and a T-cell response in BALB/C mice, significantly reducing the viral loads after challenging with CHIKV [12,13]. These results position the recombinant adenovirus as a promising vaccine candidate against CHIKV.

As extensively reported in the literature, several virus families have been shown to exploit the cellular ubiquitin-conjugating system to achieve a productive infection. The ubiquitin-conjugating system is related to several virus processes such as cell entry, genome replication, virus assembly, and release. In the search for antiviral drugs against alphaviruses, Lopez et al., 2022 [14], evaluated the effect of selective inhibitors of deubiquitinating enzymes (DUBs) on CHIKV replication. The authors found that the treatment of HEK293T, Vero-E6, and Huh-7 cells with DUB inhibitors impairs CHIKV replication due to significant protein and viral RNA synthesis deregulation. Therefore, the authors described the DUB activity as a pharmacological target for blocking CHIKV infection. Determinants of neurovirulence among alphaviruses have yet to be fully described. In this context, the determinant of virulence was identified in the macrodomain (MD) of the nonstructural protein 3 (nsP3) from CHIKV. The study compared the replication kinetics of the wild-type CHIKV with that of CHIKV containing a mutant MD site. This mutation led to either decreased ADPr binding and hydrolase activity (G32S) or increased ADPr binding with decreased hydrolase activity (Y114A) [15]. Finally, the ultrastructural morphology and mechanical properties of CHIKV were examined using atomic force microscopy and Raman spectroscopy. This analysis provided new insights into the virus’s ultrastructure at the nanoscale level [16].

In sum, these publications have significantly advanced the research on alphaviruses relevant to public health, reinforcing the need for constant monitoring, intervention strategies, and further studies to mitigate their impact. We encourage researchers, public health officials, and policymakers to continue conducting collaborative efforts, sharing knowledge, and investing in this vital area to safeguard global health.
